# Ranking of treatments in network meta-analysis: incorporating minimally important differences

**DOI:** 10.1186/s12874-025-02499-0

**Published:** 2025-03-10

**Authors:** Tristan Curteis, Augustine Wigle, Christopher J. Michaels, Adriani Nikolakopoulou

**Affiliations:** 1 Costello Medical, 50-60 Station Road, Cambridge, CB1 2JH UK; 2 Department of Statistics and Actuarial Science, University of Waterloo, Waterloo, Canada; 3 Laboratory of Hygiene, Social & Preventive Medicine and Medical Statistics, School of Medicine, Aristotle University of Thessaloniki, Thessaloniki, Greece; 4 Institute of Medical Biometry and Statistics, Medical Faculty and Medical Center, University of Freiburg, Freiburg, Germany; 5 Costello Medical, 1 New York St, Manchester, M1 4HD UK

**Keywords:** Network meta-analysis, Minimally important differences, Clinically important differences, Minimal clinically important differences, Ranking metrics, Ranking metrics, Indirect treatment comparisons

## Abstract

**Background:**

In network meta-analysis (NMA), the magnitude of difference between treatment effects is typically ignored in the calculation of ranking metrics, such as probability best and surface under the cumulative ranking curve (SUCRAs). This leads to treatment rankings which may not reflect clinically meaningful differences. Minimally important differences (MIDs) represent the smallest value in a given outcome that is considered by patients or clinicians to represent a meaningful difference between treatments. There is a lack of literature on how MIDs can be incorporated into common NMA ranking metrics such as SUCRAs to give more clinically oriented treatment rankings.

**Methods:**

Analogues to commonly available NMA ranking metrics that account for minimally important differences (MIDs) are provided. In particular, definitions are provided for MID-adjusted median ranks, MID-adjusted probability $j$ th best, MID-adjusted cumulative probability $j$ th best, and MID-adjusted SUCRA values. Since adjustment for MIDs allows for ties between treatments in a network, methods for handling ties in ranking are discussed, with it shown that the midpoint method for handling ties retains the property that the average value of all SUCRA values in a network is one half. Comparability of MID-adjusted P-scores and MID-adjusted SUCRA values is discussed, and a Bayesian software implementation of the MID-adjusted ranking metrics is provided.

**Results:**

Two real-world applications of MID-adjusted ranking metrics are presented to illustrate their use. Specifically, NMAs are conducted based on published networks on treatments for diabetes and Parkinson’s disease. To present the results, MIDs are selected from relevant literature to interpret MID-adjusted ranking metrics for these networks.

**Conclusions:**

Failure to consider MIDs when ranking treatments can lead to ranking metrics which are not clinically relevant. Our proposed MID-adjusted Bayesian ranking metrics address this challenge. Further, we show that the use of the midpoint method for addressing ties ensures comparability between standard ranking metrics and MID-adjusted ranking metrics. The methods are easily applied in a Bayesian framework using the R package mid.nma.rank.

**Supplementary Information:**

The online version contains supplementary material available at 10.1186/s12874-025-02499-0.

## Introduction

Network meta-analysis (NMA) is a commonly used method to compare the relative effects of multiple treatments when more than two treatments are available for a health condition. Popularity in use of NMA has increased significantly over the last two decades, with NMA often an important component of submission dossiers for health-technology assessment [1, 2].

Outputs of NMA include estimates of relative treatment effects such as mean differences, and odds, risk or hazard ratios, which are provided to quantify relative differences in the performance of multiple treatments. Along with estimates of the relative treatment effects, ranking metrics are often provided to support or illuminate the relationships between treatments in terms of particular outcomes. Common ranking metrics for a given outcome include the probability of a treatment being best (among the assessed treatments), the probability of being ranked in at least a given position, or the Surface Under the Cumulative RAnking curve (SUCRA) values, which represent the proportion of competing treatments that a treatment outperforms [3, 4]. More targeted ranking metrics have also been proposed to address specific clinical questions of interest [5].

Both frequentist and Bayesian methods are used as philosophical and computational frameworks for conducting NMAs, with the frequentist approach to NMA described among others by Rücker and Lumley, and Bayesian methods presented by Dias et al. [6–8]. Treatment ranking is feasible within both NMA frameworks [3, 9, 10]. Specifically, with Bayesian computation commonly conducted via Markov Chain Monte Carlo (MCMC) sampling, treatments are ranked at each Monte Carlo simulation, and ranking metrics are then generated by calculating aggregate statistics (typically the median, rather than the mean being a robust measure of central tendency that accounts for uncertainty and asymmetry in posterior distributions) over all Monte Carlo simulations [11]. Similarly, for the frequentist approach (with the exception of P-scores), ranking metrics are generated by resampling from the distribution of the relative treatment effects (assuming normality), ranking the treatments at each simulation, and generating ranking metrics as aggregates over all simulations; in this context, the mean is more common because frequentist NMA typically assumes normality of relative treatment effects, with the mean providing a straightforward average based on relative treatment effects without explicit uncertainty quantification [10–12].

In clinical trials, minimally important differences (MIDs) are often used to define statistical hypotheses, where an MID is the smallest value in a given outcome that is considered by patients or clinicians to represent a meaningful difference between treatment groups. For example, a 0.3% difference between treatment groups in hemoglobin A1c (HbA1c), an indicator of average blood glucose levels over 2–3 months prior to sampling, is considered to be clinically meaningful for patients with Type 2 diabetes [13–18]. More recently, MIDs have been suggested for use in NMAs to define hypotheses for comparisons of treatments and to define ranking metrics [5, 19–21]. MIDs are not to be confused with Minimal Important Change (MIC); this is the smallest within-patient change in an outcome measure that is considered important by patients or clinicians [22]. Although similar, the values are conceptually different and MIC is not the focus of the present work. Nevertheless, MIC and MID are commonly conflated in the literature and, when data are extracted to inform ranking statistics, care should be taken to ensure that true MID values are used [22].

Most commonly, however, ranking metrics are generated without being linked to specific clinical questions of interest. Additionally, the magnitude of difference between treatment effects is ignored in the calculation of ranking metrics (at each sampling or resampling step) [23]. In particular, MIDs are not usually considered in the calculation of differences between treatment effects. To overcome these limitations, Papakonstantinou et al. define a framework for ranking treatments in the context of specific clinical questions, with examples of considering MIDs in assessing probability of superiority between treatments, and have developed an associated R package, *nmarank,* for its implementation within a frequentist framework; [5, 24] Mavridis et al. propose the use of MIDs in defining P-scores (a frequentist analogue of SUCRAs); [20] and within a Bayesian framework, Brignardello-Petersen et al. propose accounting for MIDs when calculating the probability of a treatment being best [21].

### Study objective

Despite available literature on ranking metrics adjusting for MIDs, there is no central source for all common MID-adjusted ranking metrics. Other than MID-adjusted probability best, statistics such as MID-adjusted probabilities or cumulative probabilities of being ranked in a given position, and MID-adjusted SUCRAs have not yet been explicitly proposed. The properties of MID-adjusted SUCRAs and the relationship between MID-adjusted SUCRAs and MID-adjusted P-scores remain unclear. The interpretation of MID-adjusted ranking metrics in general could be elucidated further, and software implementation for MID-adjusted ranking metrics is not readily available. Therefore, this article aims to provide a framework for common MID-adjusted ranking metrics. Through this framework, MID-adjusted analogues to commonly available NMA ranking metrics are made available. In particular, MID-adjusted median ranks, MID-adjusted probability $j$th best, MID-adjusted cumulative probability $j$th best, and MID-adjusted SUCRA values are defined.

This article is structured as follows: common ranking metrics are presented through explicit definition of a treatment ranking function. Through the definition of a MID-adjusted ranking function, MID-adjusted ranking metrics (some of which have been proposed in other literature) are then provided. Since ties between treatments become an important feature when adjusting for MIDs, a suitable approach for handling ties is proposed. Properties of MID-adjusted SUCRA values are discussed and a clear interpretation of these MID-adjusted ranking metrics is provided. Similarities between MID-adjusted SUCRAs and MID-adjusted P-scores are also discussed; therefore, the Frequentist P-scores of Rücker and Schwarzer and their MID-adjusted extensions of Mavridis et al. are briefly presented, [10, 20]. A Bayesian software implementation for MID-adjusted ranking metrics is provided along with two real-world illustrations of MID-adjusted ranking metrics.

## Methods

In this section, we present commonly available ranking metrics, through explicitly defining a ranking function, leading to its MID-adjusted extension.

### Common ranking metrics

Suppose we are conducting an NMA for a given outcome in a network of $T$ treatments and $S$ studies. The fitted model has produced a vector of treatment effects $d$ versus a reference treatment (e.g., log odds ratios, mean differences), constituting a vector of *T* treatment effects $d$ = (${d}_{1},{d}_{2}, {d}_{3}, \dots , {d}_{T}$) relative to *T*_1_; consequently *d*_1_ is 0 since it is the effect of *T*_1_ with reference to itself. In the Bayesian sampling (Markov Chain Monte Carlo) or Frequentist resampling context, simulated values of the relative treatment effects are generated at each sampling iteration.

#### Ranking function

For each treatment $k$, a rank can be assigned according to the size of its treatment effect relative to all other treatments in the network. For a positively oriented outcome (i.e., where higher values or more events are better), we define the rank of treatment $k$ as:

1 $$R(k)=T-\sum\limits^{T}_{j=1,j\ne k}\mathbbm{1}_{\{{d_\text{k}-d_{j}\ge 0}\}},$$

where ${\mathbbm{1}}_{\left\{\text{x}\right\}}$ takes value 1 when $x$ is true, and 0 otherwise. In other words, the rank of treatment $k$ is calculated as the number of treatments in the network minus the number of treatments to which treatment $k$ is superior. This step (or an equivalent) is typically implicit within available software for ranking in NMA.

For example, for the number of treatments $T=4$ and assuming that a higher outcome is better with the treatment effect vector $d=(0,\ 1,\ 2,\ 3)$, the vector indicating the number of superiorities would be $\left(0,\;1,\;2,\;3\right)$, and the vector of ranks $\left(4-0,\;4-1,\;4-2,\;4-3\right)=\left(4,\ 3,\ 2,\ 1\right)$. It is based on the above (or an equivalent) ranking function that treatments are commonly ranked, and their subsequent ranking metrics (e.g., probability best, SUCRAs) are defined. For $T$ treatments, treatment $k$ ranked ${j}^{th}$ would be superior to $T-j$ other treatments.

Similarly, the equivalent ranking function for a negatively oriented outcome could be defined as:

$$R(k)=T-\sum\limits^{T}_{j=1,j\ne k}\mathbbm{1}_{\{d_{j}-d_{k}\ge 0\}},$$

where the indices for $j$ and $k$ have been reversed.

#### Ranking Metrics

It is implicitly based on a ranking function alone that median ranks, probability best, probability ${j}^{th}$ best, probability at least ${j}^{th}$ best, and SUCRA values are defined. For example, for treatment $k$,

2 $${P}_{best}\left(k\right)=P\left(R\left(k\right)=1\right),$$

3 $${P}_{{j}^{th}}\left(k\right)=P\left(R\left(k\right)=j\right),$$

and the cumulative probability that treatment $k$ is at least ${j}^{th}$ is given by:

4 $$CP(k,j)=\sum^{j}_{i=1}P(R(k)=i).$$

In the sampling context, these individual probabilities are easily calculated: the proportion of times (across all sample iterations) that treatment $k$ is ranked ${j}^{th}$ provides the probability that treatment $k$ is ranked ${j}^{th}$. Median ranks are also readily available and are calculated as the median across all sampled ranks for each treatment. Averaging over all cumulative probabilities provides a SUCRA value for each treatment $k$, as follows:

5 $$SUCRA_{k}=\frac{1}{T-1}\sum\limits^{T-1}_{r=1}\sum\limits^{r}_{j=1}P(R(k)=j),$$

which can be interpreted as the proportion of treatments in the network that treatment $k$ performs better than.

### MID-Adjusted ranking metrics

#### MID-Adjusted ranking function

Consider now an adaptation of the above ranking function, where treatments are only considered to be superior/inferior to each other if the difference between them is greater than or equal to a minimally important difference, MID. The positively oriented ranking function could be defined as:

6 $${R}_{MID}\left(k\right)=T-{\sum }_{j=1, j\ne k}^{T}\mathbbm{1}_{\left\{{d}_{k}-{d}_{j}\ge MID\right\}.}$$

This has the interpretation that the rank of treatment $k$ is determined by the proportion of competing treatments that it outperforms by at least a value of MID. The term ‘$d_k-d_j\geq MID$’, on which superiority of treatment $k$ over $j$ is determined, has also been used to define MID-adjusted probabilities between two (or more) treatments, as presented by Papakonstantinou et al. [5]. We call this the ‘decision rule’.

#### Handling of ties in ranks

In the case of $MID=0$, i.e., equation [1], the probability of a tie occurring in the sampling context is effectively zero as this implies identical sampled relative effects. Even if two treatments have the same treatment effect (e.g., hazard ratio) reported in each of their studies, ties in ranking between the two treatments at a given sampling iteration are therefore almost impossible. As such, the approach to handling ties has been safely ignored in NMA literature to date. However, in the case of an MID value greater than zero, ties in ranking between treatments may occur, and this must be accounted for.

Ties may be accounted for by replacing each tied value with the minimum (commonly used in sports), the maximum (employed in the definition of ${R}_{MID}$ in equation), or the midpoint of the rankings that would have been realised. For example, consider a treatment effect vector ${\text{d}}=\left(0,\;0.3,\;0.5,\;5\right)$ where all treatments have a different effect (no direct ties), and an MID of 0.25 is established. Treatments ${d}_{2}$ and ${d}_{3}$ become equivalent as they are within 0.25 units of one another, such that ranks 2 and 3 are tied. The minimum approach therefore yields ranks of $\left(4,\;2,\;2,\;1\right)$, the maximum approach yields $\left(4,\;3,\;3,\;1\right)$ and the midpoint approach yields $\left(4,\;2.5,\;2.5,\;1\right)$.

#### Average SUCRA values and MID-adjusted ranking metrics

Before proceeding to define MID-adjusted ranking metrics, we note that a desirable property of SUCRA values is that their average across a network is one half [10]. This fact can provide an orientation in interpreting SUCRA values for a given network. We provide a proof (Supplementary material 1) that this fact holds in the case of allowing for ties between treatments when the midpoint method for handling ties is selected. In the case of the minimum or maximum value approach to handling ties, the average SUCRA value depends on the number of ties and their position. Given this property for the midpoint method of handling ties, we present MID-adjusted ranking metrics below which apply the midpoint method for handling ties.

The ranking function for the midpoint method can be defined as follows. Suppose there are $L$ rank locations where there are ties, with ${m}_{l}$ ($l=1,\dots ,L$) the number of treatments that are tied at a given rank; then,

7 $${R}_{MID}\left(k\right)=\left\{\begin{array}{c}T-\frac{m_l-1}{2}-\sum\limits^{T}_{j=1,j\ne k}\mathbbm{1}_{\{{d_\text{k}-d_{j}\ge MID}\}},\;if\;m_l-1\;treatments\;tied\;with\;teatment\;k,\\ T-\sum\limits^{T}_{j=1,j\ne k}\mathbbm{1}_{{\{\text{d}_{k}-d_{j}\ge MID}\}},\;if\;no\;treatments\;tied\;with\;treatment\;k. \end{array}\right.$$

Note that when no treatments are tied with k (${m}_{l}-1 = 0$), the first case reduces to the second case. Additionally, for each sampling iteration, if the number of treatments tied with treatment k is even, the MID-adjusted rank will end in 0.5, while if there is an odd number of treatments tied with k, the MID-adjusted rank will take an integer value. This odd–even rule does not apply to overall ranking metrics, only to the ranking function employed at each iteration.

${R}_{MID}$, as defined in [7] is applied to calculate MID-adjusted ranks in all samples of relative effects. MID-adjusted mean and median ranks can then be calculated across all samples. Note that the value of ${R}_{MID}\left(k\right)$ within each sample can take values of 1, 1.5, 2, etc. while ranking metrics such as the MID-adjusted mean ranks, and median ranks calculated using certain approaches, may take on other values since they are a summary of the ${R}_{MID}\left(k\right)$ over all samples.

MID-adjusted median ranks are the median rankings of each treatment, acknowledging MIDs between treatments. Since ties are possible, and the midpoint method is employed, rankings of 1st, 1.5th, 2nd, 2.5th etc. are possible. Therefore, the best treatment may be ranked first, or for example, 1.5th in the case of a tie between two treatments for the winning position. As such, there are two alternative definitions of the probability of being best. First, the equation

8 $${P}_{best}\left(k\right)=P\left({R}_{MID}\left(k\right)=1\right),$$

provides the probability that a treatment is superior to all others by at least an MID value. This is the quantity calculated in Brignardello-Petersen et al. (albeit they take the minimum approach to handling ties, rather than the midpoint) [21]. Additionally, we could calculate an MID-adjusted probability best as:

9 $${P}_{best}\left(k\right)=P\left({R}_{MID}\left(k\right)=\text{min}({R}_{MID}\left(1\right), {\dots ,R}_{MID}(T)\right),$$

Which is the probability of being within an MID of the best treatment (i.e., best or equal best).

Furthermore, through employing ${R}_{MID}$ of equation [7] to introduce an MID and handle ties in equation [3], it is possible to calculate the MID-adjusted probability $j$th best, which is the probability of being superior by an MID value to $T-j$ treatments. Similarly, using ${R}_{MID}$ in equation [4], the cumulative MID-adjusted probability of being ranked at least $j$th best can be calculated. This is the probability of being ranked at least $j$th or better in terms of MID-superiorities. Additionally, through specifying ${R}_{MID}$ in equation [5], MID-adjusted SUCRA values can be calculated. These can be viewed as the inversely scaled average rank of a given treatment, where rank is determined by considering an MID. The specific j of interest may be context dependent (for example, if budget is only available for three competing treatments, then the probability of being ranked at least third best is of interest). As a more general approach, we suggest generating the plots which show all possible j values – rather than selecting a specific j for interpretation necessarily (see Supplementary material 2).

For completeness, the MID-adjusted ranking metrics, which employ the ranking function of equation [7], are provided in Table 1. As noted above, these MID-adjusted ranking metrics can be interpreted in a similar way to their unadjusted analogues, with the addition that they consider MIDs between treatments. Based on the description of the ranking function [7], it is clear that treatment ranks are related directly to the number of MID-superiorities. Furthermore, since the midpoint method for handling ties is employed, $j$ no longer only takes integer values and can be any value in the set 1, 1.5, 2, 2.5,…, T.

**Table 1:** Formulae for MID-Adjusted Ranking Metrics

Metric	MID-Adjusted Formula
MID-Adjusted Probability Best	${P}_{best}\left(k\right)=P\left({R}_{MID}\left(k\right)=1\right)$
	$${P}_{best}\left(k\right)=P\left({R}_{MID}\left(k\right)=\text{min}({R}_{MID}\left(1\right), {\dots ,R}_{MID}(T)\right)$$
MID-Adjusted Probability j^th^ best	${P}_{{j}^{th}}\left(k\right)=P\left({R}_{MID}\left(k\right)=j\right)$
MID-Adjusted Cumulative Probability at Least j^th^ best	$CP(k,j)=\sum\limits^{j}_{i=1}P(R_{MID}(k)=i).$
MID-Adjusted SUCRA	$SUCRA_{k}=\frac{1}{T-1}\sum\limits^{T-1}_{r=1}\sum\limits^{r}_{j=1}P(R_{MID}(k)=j)$

### P-scores

A frequentist analogue of SUCRA values known as P-scores, which need not be calculated in a sampling context, is defined as:

$$PScore_k=\frac1{T-1}\sum_{j,j\neq k}^T\Phi\left(\frac{{\widehat{d_{k}}}-{\widehat{d_{j}}}}{s_{kj}}\right),$$

where $\Phi$ is the standard cumulative normal distribution and where ${s}_{kj}$ is the standard error of the difference ${d}_{k}-{d}_{j}.$ [10] Mavridis et al. incorporated an MID to extend this to: [20].

10 $$PScor{e}_{k}=\frac{1}{T-1}\sum\limits^{T}_{j,j\ne k}\Phi \left(\frac{\widehat{d_{k}}-\widehat{d_{j}}-MID}{s_{kj}} \right).$$

### P-scores and SUCRA values

As derived in Rücker 2015, P-scores have been shown to be equal to SUCRA values when probabilities are considered to be known [10]. However, due to the use of the midpoint method to handle ties in this paper, we expect deviations in estimated values between the MID-adjusted P-scores of Mavridis et al. [20] and the MID-adjusted SUCRAs of this article. This is because the average of the Mavridis MID-adjusted P-scores is not 0.5, whereas the average of MID-adjusted SUCRAs is 0.5 (in the case of the midpoint method). Additionally, by definition, the MID-adjusted P-scores do not need to consider handling of ties, unlike MID-adjusted SUCRAs. MID-adjusted P-scores are a direct calculation of a probability based on an effect size and reflect the proportion of times a treatment outperforms any other by an MID value and exclude the proportion of times they are equal by an MID value. As such, in calculation of P-scores, there is no method required for handling ties. The definition of an MID-adjusted SUCRA is based on the cumulative probability of ranking in a given position, for which handling of ties is required in the case of an MID. However, despite the values being different for the reasons explained above, we expect both MID-adjusted SUCRAs and MID-adjusted P-scores to draw similar implications in terms of comparative positioning of treatments.

Finally, given this lack of exact correspondence with MID-adjusted P-scores, MID-adjusted SUCRAs no longer retain the unadjusted SUCRA interpretation of ‘the proportion of treatments that a treatment outperforms’. An interpretation of the MID-adjusted SUCRA values is the inversely scaled average rank of a treatment, where the ranking considers MIDs, and ties are treated as defined in the ranking function in Equation [7].

### Software implementation

As an extension to the *multinma* package in software R, [25] we have developed an associated R package, *mid.nma.rank,* which provides a Bayesian implementation of the methods described in this article [26]. The package *mid.nma.rank* adapts existing ranking functionality within the *multinma* package to produce the MID-adjusted ranking metrics described in this article. The package and its code, as well as code to implement examples shown below, can be accessed through the web link provided in the Data Availability section.

## Results

Here, as an illustration of the above methods, we reanalysed two datasets via the R package *multinma* (which internally uses the software Stan) [25]. For both datasets, a Bayesian model with a normal distribution was fitted to mean treatment differences. We then used our package *mid.rank.nma* to generate MID-adjusted ranking metrics [26]. Default vague priors were used, with a half-normal distribution for the between-study standard deviation parameter. For both analyses, four chains each with 25,000 iterations burn-in and an additional 25,000 iterations for inference were run.

### Parkinsons example

**Fig. 1:**
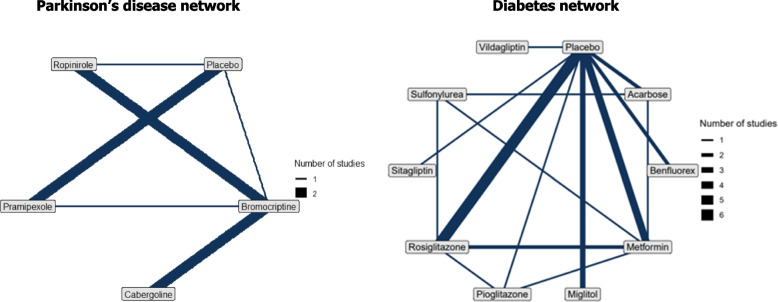
Network diagram: Parkinson’s disease and diabetes NMA

Consider the following example of a network of treatments for Parkinson’s disease, sourced from NICE DSU TSD 2 [27]. Seven studies reported on five treatments, where patients were given dopamine agonists as adjunct therapy or placebo. The mean reduction in ‘off’ time, interpreted as the latency of the return of symptoms (motor or nonmotor) between medication doses, was investigated. The network diagram is shown in Fig. 1, with mean differences between treatments in off-time reported in Table 2. Despite some differences across the literature, we consider one hour to be an MID in off-time [28, 29]. We note that although this value was originally reported under the term ‘MID’, its definition more closely fits that of MIC, i.e. within-patient rather than between-treatment difference. Nevertheless, the value has subsequently been used as an MID to inform work including clinical trials, and is similar to other related values used to inform treatment difference non-inferiority margins for treatment comparisons [28, 30, 31]. Consequently, and as the value is used principally as an illustration of statistical methodology and not to influence the field of Parkinson’s disease treatment, we considered the value of 1 h a suitable MID for off time in the present work.

**Table 2:** Parkinson’s NMA ranking metrics: with and without MID adjustment (MID = 1)

	*Median Rank*	*MID-Median* *Rank*	*SUCRA*	*MID-SUCRA*	*P Best*	*MID-P Best*	*MID-P Best or Equal Best*
*Placebo*	5	3.5	0.09	0.29	0.00	0.00	0.00
*Pramipexole*	1	1	0.99	0.95	0.96	0.82	0.99
*Ropinirole*	4	3.5	0.37	0.37	0.00	0.00	0.09
*Bromocriptine*	3	3.5	0.38	0.38	0.00	0.00	0.09
*Cabergoline*	2	3.5	0.68	0.50	0.04	0.00	0.17

P Best is the probability of being best – ignoring MIDs between treatments; MID P Best is the probability of being superior to all other treatments by at least an MID; MID-P Best or Equal Best is the probability of being best, or within an MID of the best treatment (i.e., equal)

**Fig. 2:**
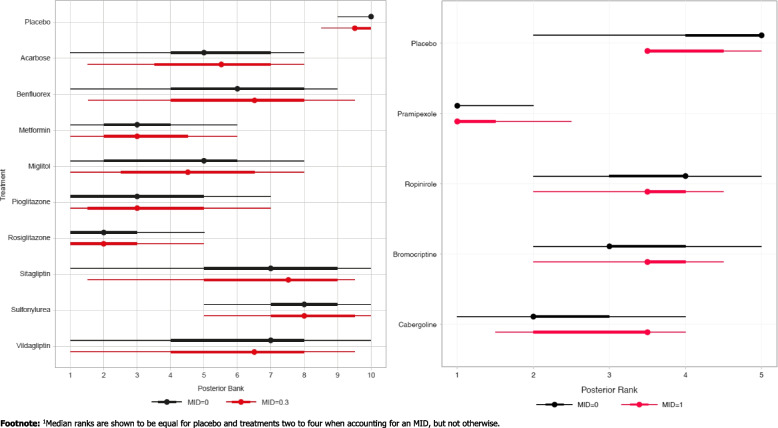
Left: Diabetes NMA: Distribution of ranks, with and without MID adjustment (MID = 0.3). Right: Parkinson’s NMA: Distribution of ranks, with and without MID adjustment (MID = 1 h). The narrow error bars represent 95% and the thicker bars 66% credible intervals

**Fig. 3:**
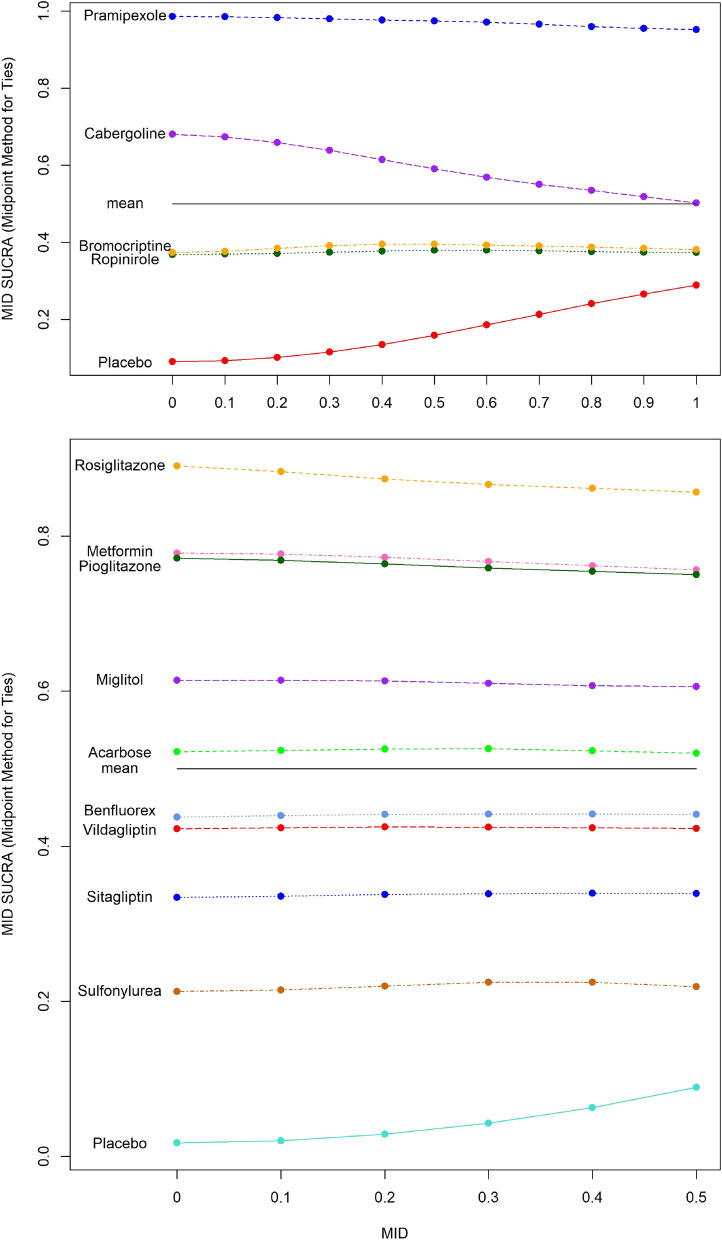
Top: Parkinson’s NMA: MID-Adjusted SUCRA Sliding Scale from MID = 0 to MID = 1. Bottom: Diabetes NMA: MID-adjusted SUCRAs sliding scale from MID = 0 to MID = 0.5

Given the small number of studies per treatment contrast (thereby complicating estimation of the between-study standard deviation parameter), the fixed effects model was selected. The distributions of ranks with and without MID adjustment are displayed in Fig. 2. Notably, when considering an MID of 1 h, cabergoline, bromocriptine, and ropinirole are considered to be no better than placebo in terms of median rank. Only pramipexole appears to result in a clinically relevant treatment effect compared to placebo when we consider an MID of 1 h. If the MID were not considered, researchers may conclude that each treatment has a different ranking, although the differences in ranks may not be associated with a clinically meaningful difference. The MID approach thus provides a ranking with a more clinically oriented interpretation. Figure 3 presents MID-adjusted SUCRA values and their mean, with MID adjustments ranging from 0 to 1 h. As the MID increases from zero, the SUCRA values for cabergoline, bromocriptine, ropinirole and placebo become closer together, whereas the SUCRA value for pramipexole remains close to 1. This reflects the fact that there is less reason to prefer one of cabergoline, bromocriptine, ropinirole or placebo as the MID increases, but pramipexole remains superior to the others even when an MID of 1 h is considered. We also demonstrate that the mean of the MID-adjusted SUCRAs is 0.5 for all values of the MID.

Further results are provided in Table 2 and Supplementary material 2, including MID-adjusted (cumulative) probability of ranking in a given position, and a comparison of MID SUCRA values and MID P-scores. We note that although the exact values of the SUCRAs differ substantially from those of the P-scores, the ordering of the treatments that the MID-adjusted SUCRA values suggest is the same as that suggested by the MID-adjusted P-scores.

### Diabetes example

For an example in diabetes, we take the Senn 2013 dataset, available in the package *netmeta (also in mid.nma.rank)*, which was originally sourced from Senn 2013 [9, 32]. The dataset consists of 26 studies on 10 treatments (including placebo), and the outcome under consideration is the mean difference in change from baseline in mean HbA1c, a measure of blood sugar attached to haemoglobin and an indicator of average blood sugar levels in preceding weeks. The network diagram is shown in Fig. 1. A difference between groups of 0.3 in % HbA1c is considered to be clinically meaningful for patients with type 2 diabetes [13–15]. Importantly, this differs from an MIC value of 0.5% reported in the literature, which was not used here [16–18].

Due to the number of studies informing each treatment contrast, the random effects model was preferred over the fixed effects model. MID-adjusted ranking metrics and their unadjusted analogues are displayed in Table 3. A plot of ranking with and without accounting for MID is shown in Fig. 2 and a plot of SUCRA values over a range of MID values is shown in Fig. 3. The primary finding from this diabetes example is that the ranking metrics are reasonably similar with and without adjustment for an MID (i.e., reasonably robust with reference to MID adjustment). Further plots are provided in Supplementary material 2.

**Table 3:** Diabetes NMA ranking metrics: with and without MID adjustment (MID = 0.3)

	*Median Rank*	*MID-Median* *Rank*	*SUCRA*	*MID-SUCRA*	*P Best*	*MID-P Best*	*MID-P Best or Equal Best*
*Placebo*	10	9.5	0.02	0.04	0.00	0.00	0.00
*Acarbose*	5	5.5	0.52	0.53	0.03	0.01	0.10
*Benfluorex*	6	6.5	0.44	0.44	0.03	0.02	0.07
*Metformin*	3	3	0.78	0.77	0.13	0.05	0.34
*Miglitol*	5	4.5	0.61	0.61	0.09	0.05	0.19
*Pioglitazone*	3	3	0.77	0.76	0.23	0.12	0.41
*Rosiglitazone*	2	2	0.89	0.87	0.42	0.24	0.63
*Sitagliptin*	7	7.5	0.33	0.34	0.03	0.02	0.05
*Sulfonylurea*	8	8	0.21	0.22	0.00	0.00	0.00
*Vildagliptin*	7	6.5	0.42	0.43	0.05	0.03	0.10

P Best is the probability of being best – ignoring MIDs between treatments; MID P Best is the probability of being superior to all other treatments by at least an MID; MID-P Best or Equal Best is the probability of being best, or within an MID of the best treatment (i.e., equal)

## Discussion

The ranking of treatments in NMAs has wide societal implications. In particular, the ranking of an emerging treatment in terms of efficacy and safety contributes (along with other manifold considerations such as cost) to its positioning among existing treatment options and therefore its accessibility to patients, propensity of usage by physicians, and associated market share for pharmaceuticals. As illustrated in our Parkinson’s example, failure to consider MIDs in ranking treatments can lead to less clinically relevant treatment rankings, while the MID approach lends itself to a clinically orientated interpretation. In this way, the MID ranking metrics offer an alternative perspective, which can supplement traditional ranking metrics that do not consider an MID. Although median ranks have been presented in this work (as we provided a Bayesian implementation), mean ranks could also be calculated.

Specifying an MID is commonplace when defining hypotheses in clinical trials. Uhlmann et al. extended this principle to NMAs, presenting an MID-based hypothesis framework in the NMA context to gauge the superiority or noninferiority of treatments [19]. MIDs are therefore conspicuous by their absence in commonly reported NMA ranking metrics. Recent Joint Clinical Assessment (JCA) guidelines, which adopt the EUnetHTA guidelines, note that in population-adjusted analyses, or if there is a risk of bias or confounding in a network, specifying a threshold away from the typical line of no treatment effect (i.e., an MID) is recommended [33, 34].

Interestingly, although dependent on the magnitude of the MID, each MID-adjusted ranking statistic is more likely to evaluate treatments as equivalent (i.e., no MID difference between treatments) than the corresponding conventional NMA treatment ranking, which does not take into account an MID value (or which sets the MID to zero). In other words, treatments are more likely to be considered comparable when considering MIDs. Conversely, this means that if an MID is not employed for ranking metrics and the focus of interpretation is solely on the ranking statistics without consideration of estimated relative effects, treatments are likely to be interpreted as being more dissimilar than is clinically accurate. As illustrated by our examples in diabetes and Parkinson’s disease, the extent to which this is the case will depend on both the degree of difference between treatments and the magnitude of the MID. The selection of an MID value involves a degree of subjectivity. This is important to consider since some treatment differences may be very close to, but slightly below, the selected MID threshold, which could be disadvantageous to patients who may prefer to opt for the treatment with the greater effect of the two. A more nuanced approach might involve MID-based ranking to identify and equate the subset of treatments that differ from placebo less than the MID, and then to implement standard ranking methods for those that exceed it. Further research to investigate hybrid approaches such as this and their relative merits in terms of treatment selection and disadvantages in terms of subjective input from clinicians and researchers, may be of value.

An expected challenge in the uptake of MIDs in the ranking of treatments from an NMA is the effort required to derive or define the MID. As per the examples in this article, the specific MID may be based on clinical opinion or published literature, which may or may not be available for the outcome or scale of outcome [e.g., log odds ratio] in question. Further, it is important to distinguish MIDs from MICs, especially given some conflation of the two concepts in the literature. The German HTA guidelines note that there is no established standard method to estimate MIDs and that there is some variability in estimated MIDs across different methodologies [29]. With the midpoint method for handling ties, our ranking function loses the interpretation of that of ‘standard competition ranking’ used in sports, which is undesirable. However, the midpoint approach retains the property that the average of all SUCRA values is equal to one half, which makes it possible to compare them with unadjusted SUCRAs. On balance, the midpoint approach was consequently considered preferable.

Finally, if the probability of holding a rank other than 1 is of interest, selection of appropriate or useful values of j is required and may become unintuitive in the case of non-integer values of j in the case of ties. An alternative approach might consider the probability of treatment (k) ranking among the jth best treatments, where j ranges from 1 to J (with J being the total number of distinct treatment groups separated by at least an MID value). This approach would essentially provide the cumulative probability that a treatment is within the top j groups, which might align more closely with clinical decision-making needs, especially when non-integer ranks are less meaningful in practice. Such approaches might be recommended for future work building on the methods presented here.

## Conclusion

In this article, we provide an approach for deriving MID-adjusted treatment ranking metrics for NMA. To our knowledge, existing MID-adjusted ranking metrics have focused only on probability best, P-scores, and an example of ranking between two treatments when an MID is under consideration [5, 20, 21]. We provide MID-adjusted versions of median ranks, probability best, probability j^th^, cumulative probability j^th^ and SUCRA values. We also show that by using the midpoint method for ranking, the MID-adjusted SUCRA values retain the property that their average is 0.5 (as per regular SUCRA values without MID adjustment), facilitating their interpretation. We additionally comment on the comparability of MID-adjusted P-scores and MID-adjusted SUCRAs. Furthermore, although our suggested MID-adjusted statistics could be estimated in both frequentist (resampling) and Bayesian (sampling) contexts, we provide a Bayesian software implementation via the R package *mid.nma.rank* (available on GitHub), which is an add-on to the existing Bayesian NMA R package *multinma*. Finally, we illustrated the use of these MID-adjusted ranking metrics in two clinical examples.

In conclusion, integrating MIDs into NMA ranking metrics as an adjunct to existing approaches and methodology represents an advancement in the clinical interpretability of NMA results. Considering clinically relevant differences in ranking metrics allows for the creation of useful and interpretable treatment rankings to enrich existing methodologies. The practical application of these methods in the fields of diabetes and Parkinson's disease highlights their utility and relevance in real-world scenarios. Furthermore, we provide a Bayesian software package to encourage the adoption of this approach, which promises improved decision making from NMAs.

## Supplementary Information

Supplementary Material 1.

Supplementary Material 2.

## Data Availability

The associated analysis code and datasets for this paper are available on GitHub: https://github.com/tristancurteis/mid.nma.rank.

## Abbreviations

MID: Minimally important difference
NMA: Network meta-analysis
JCA: Joint Clinical Assessment
EUnetHTA: European Network for Health Technology Assessment
SUCRA: Surface Under the Cumulative Ranking Curve
MCMC: Markov Chain Monte Carlo
NICE: National Institute for Health and Care Excellence
DSU: Decision Support Unit
TSD: Technical Support Document
DIC: Deviance Information Criterion
