# Supplementary material for: Ranking of treatments in network meta-analysis: incorporating minimally important differences
Source: BMC Med Res Methodol. 2025 Mar 10;25:67. doi: 10.1186/s12874-025-02499-0 (PMC11892231; doi:10.1186/s12874-025-02499-0)
Supplement: Supplementary file 1 — Supplementary Material 1. [file 12874_2025_2499_MOESM1_ESM.docx]

# SUPPLEMENTARY MATERIAL 1

## Average SUCRA values

Author: Augustine Wigle

It has been stated that the average SUCRA value among $T$ treatments is equal to 0.5. That is,

$\frac{1}{T}\sum_{i=1}^{T} SUCRA(i)=\frac{1}{2}$.

For completeness, we provide a proof when no ties are permissible below and then extend the proof to the case in which ties are permissible and handled via the midpoint method.

### Proof With No Ties

The SUCRA can be expressed in terms of the expected rank of treatment $i$ as follows:

$$SUCRA\left( i \right)=\frac{T-E(rank(i))}{T-1}.$$

Therefore,

$$\frac{1}{T}\sum_{i=1}^{T} SUCRA\left( i \right)= \frac{1}{T}\sum_{i=1}^{T} \frac{T-E\left( rank\left( i \right) \right)}{T-1}$$

$$= \frac{1}{T}\frac{1}{T-1}\left( \sum_{i=1}^{T} T- \sum_{i=1}^{T} E\left( rank\left( i \right) \right) \right)$$

$$=\frac{1}{T}\frac{1}{T-1}\left( T^{2}-E\left( \sum_{i=1}^{T} rank(i) \right) \right)$$

$$=\frac{1}{T}\frac{1}{T-1}\left( T^{2}-\frac{1}{2}T(T+1) \right)$$

**( 11 )**

$$=\frac{1}{T}\frac{1}{T-1}\left( \frac{1}{T}T^{2}-\frac{1}{2}T \right)$$

$$=\frac{1}{2}\left( \frac{T(T-1)}{T(T-1)} \right)$$

$=\frac{1}{2}$.

We note that in equation **( 11 )** we used the fact that, if there are no ties, the sum of all the ranks is the sum of all possible positions and,

$\sum_{i=1}^{T} rank\left( i \right)= \sum_{i=1}^{T} i= \frac{1}{2}T(T+1)$.

**( 12 )**

### Proof with the midpoint method for ties

Now, we assume that if ties are allowed and tied treatments are assigned ranks corresponding to the midpoint method, equation **( 12 )** holds, and as such, the average SUCRA value is still one half.

Let us define the way in which ties are handled via the midpoint method. Suppose that $m$ treatments are tied for rank $v.$ For example, for treatment effects (2, 3.5, 3.5, 6), the tied position is $v=2$. Then, rather than taking the minimum approach of $rank\left( i \right)=v= 2$ for both tied treatments and instead using the midpoint approach, we define the rank of tied treatments as:

$$rank\left( i \right)=v+ \frac{m-1}{2},$$

That is, the average of all ranks$v, v+1, \ldots v+m-1.$

Now, suppose there are $L$ rank locations where there are ties. Let $v_{l}$ with $l=1, \ldots, L$ represent the location of the treatment effect vector where ties occur and let $m_{l}$ with $l=1,\ldots,L$ be the number of treatments that are tied at rank $v_{l}$. For treatments tied at rank $v_{l}$ based on the minimum approach, the corresponding midpoint rank is given by:

$$v_{l}+ \frac{m_{l}-1}{2}.$$

To assess the validity of equation **( 12 )** in the case of ties handled via the midpoint method, the sum of realised ranks can be written as the sum of ranks with no ties plus the sum of ranks of tied treatments. That is, as

$$\sum_{i=1}^{T} rank\left( i \right)=\left( sum of ranks with no ties \right)+\left( sum of ranks of tied treatments \right).$$

**( 13 )**

Since there are $m_{l}$ ties taking the midpoint value, the sum of ranks of tied treatments can be written as:

$$\sum_{l=1}^{L} m_{l}\left( v_{l}+ \frac{m_{l}-1}{2} \right),$$

Additionally, the sum of ranks of treatments without ties can be written as the sum of all possible ranks (the sum of all integer values) minus the sum of the ranks that would have been realised had there not been ties. When there are $m_{l}$ ties at position $v_{l}$, an expression for the affected would have been realised ranks is $v_{l}+a-1$, where $a=1, \ldots, m_{l}.$ That is as:

$$\sum_{i=1}^{T} i- \sum_{l=1}^{L} \sum_{a=1}^{m_{l}} \left( v_{l}+a-1 \right).$$

Therefore, equation **( 13 )** can be rewritten as

$$\sum_{i=1}^{T} rank\left( i \right)= \sum_{i=1}^{T} i- \sum_{l=1}^{L} \sum_{a=1}^{m_{l}} \left( v_{l}+a-1 \right)+ \sum_{l=1}^{L} m_{l}\left( v_{l}+ \frac{m_{l}-1}{2} \right)=\frac{1}{2}T\left( T+1 \right)-\sum_{l=1}^{L} \left( m_{l}v_{l}+ \frac{1}{2}m_{l}\left( m_{l}+1 \right)-m_{l} \right)+ \sum_{l=1}^{L} \left( m_{l}v_{l}+ \frac{1}{2}m_{l}\left( m_{l}+1 \right)-m_{l} \right)=\frac{1}{2}T\left( T+1 \right)+0$$

$$=\frac{1}{2}T\left( T+1 \right).$$

Therefore, equation **( 11 )** holds in the case of the midpoint method for ties, and the average SUCRA value is still one half.

In the case of the ‘minimum’ and ‘maximum’ rather than the midpoint method to handle ties, for $m_{l}$ties at location $v_{l}$, the minimum and maximum ranks are $rank\left( i \right)=v_{l}$ and $rank\left( i \right)=v_{l}+m_{l}-1$, respectively. And it can be shown that the average SUCRA value depends on $v_{l}$ and $m_{l}$, the number of ties and their respective locations.
