# Supplementary material for: Ranking of treatments in network meta-analysis: incorporating minimally important differences
Source: BMC Med Res Methodol. 2025 Mar 10;25:67. doi: 10.1186/s12874-025-02499-0 (PMC11892231; doi:10.1186/s12874-025-02499-0)

# SUPPLEMENTARY MATERIAL 2

Additional results for the illustrative examples are provided in this supplementary material. NMA models fitted via the multinma package used their default priors, i.e., half_normal (location = 0, scale = 5), for between-study heterogeneity.

Supplementary Table 1. Parkinson’s NMA: Comparison of MID-adjusted SUCRA and MID-adjusted P-scores

|  | P-score (MID=0) | SUCRA (MID=0) | P-score (MID=1) | MID-SUCRA (MID=1) |
| --- | --- | --- | --- | --- |
| Placebo | 0.09 | 0.09 | 0.00 | 0.29 |
| Pramipexole | 0.99 | 0.99 | 0.73 | 0.95 |
| Ropinirole | 0.37 | 0.37 | 0.04 | 0.37 |
| Bromocriptine | 0.38 | 0.38 | 0.04 | 0.38 |
| Cabergoline | 0.68 | 0.68 | 0.10 | 0.50 |

Supplementary Table 2. Diabetes NMA: Comparison of MID-adjusted SUCRA values and P-scores

|  | P-score (MID=0) | SUCRA (MID=0) | P-score (MID=0.3) | MID-SUCRA (MID=0.3) |
| --- | --- | --- | --- | --- |
| Placebo | 0.01 | 0.02 | 0.00 | 0.04 |
| Acarbose | 0.52 | 0.52 | 0.33 | 0.53 |
| Benfluorex | 0.44 | 0.44 | 0.26 | 0.44 |
| Metformin | 0.78 | 0.78 | 0.54 | 0.77 |
| Miglitol | 0.61 | 0.61 | 0.40 | 0.61 |
| Pioglitazone | 0.77 | 0.77 | 0.54 | 0.76 |
| Rosiglitazone | 0.89 | 0.89 | 0.64 | 0.87 |
| Sitagliptin | 0.33 | 0.33 | 0.19 | 0.34 |
| Sulfonylurea | 0.21 | 0.21 | 0.11 | 0.22 |
| Vildagliptin | 0.42 | 0.42 | 0.25 | 0.43 |

Supplementary Figure 1 Parkinson NMA: Forest plot: mean difference in off-time, treatment versus placebo (fixed effects NMA). The narrow error bars represent 95% and the thicker bars 66% credible intervals


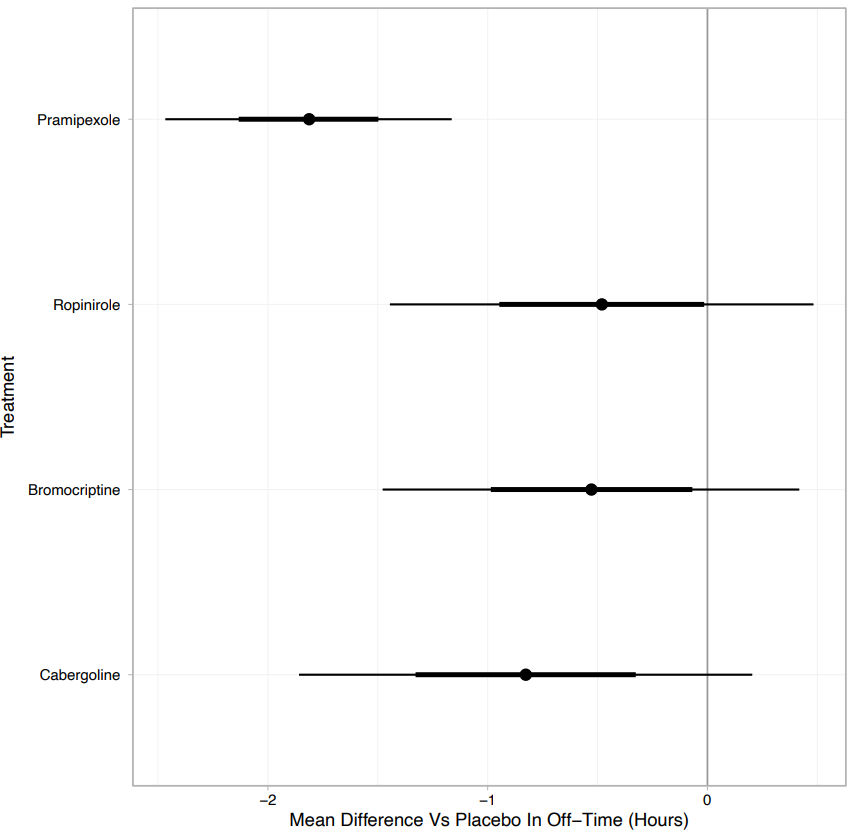


Supplementary Figure 2. Parkinson’s NMA: MID-adjusted probability j^th^ best (MID=1), where 1 is the best rank


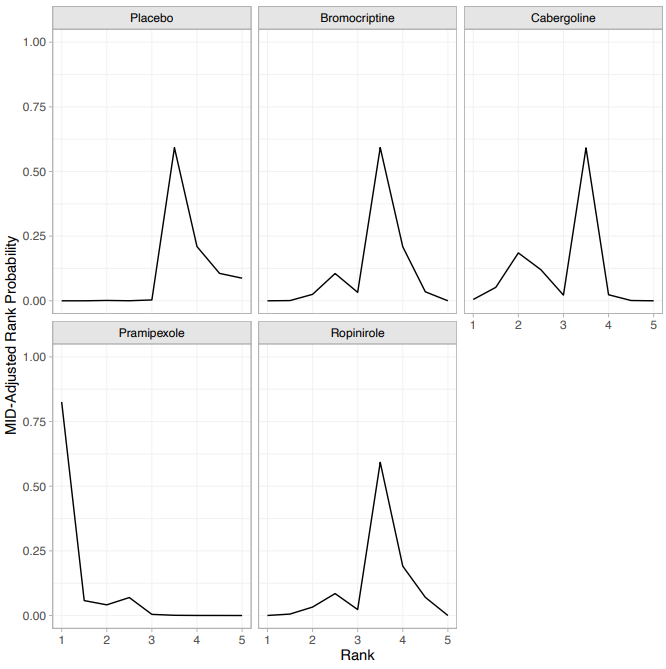


Supplementary Figure 3. Parkinson’s NMA: MID-adjusted cumulative probability j^th^ best (MID=1), where 1 is the best rank


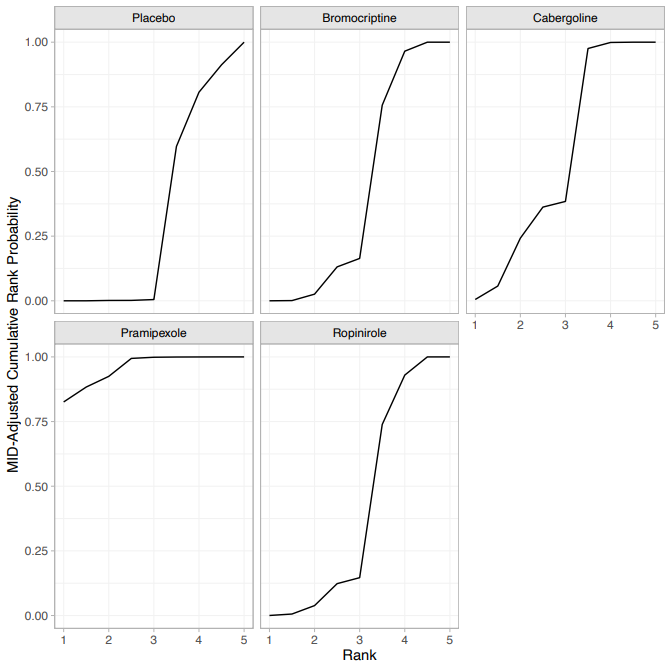


Supplementary Figure 4. Diabetes NMA: Forest plot: mean difference in HbA1c, treatments versus placebo (random effects NMA). The narrow error bars represent 95% and the thicker bars 66% credible intervals


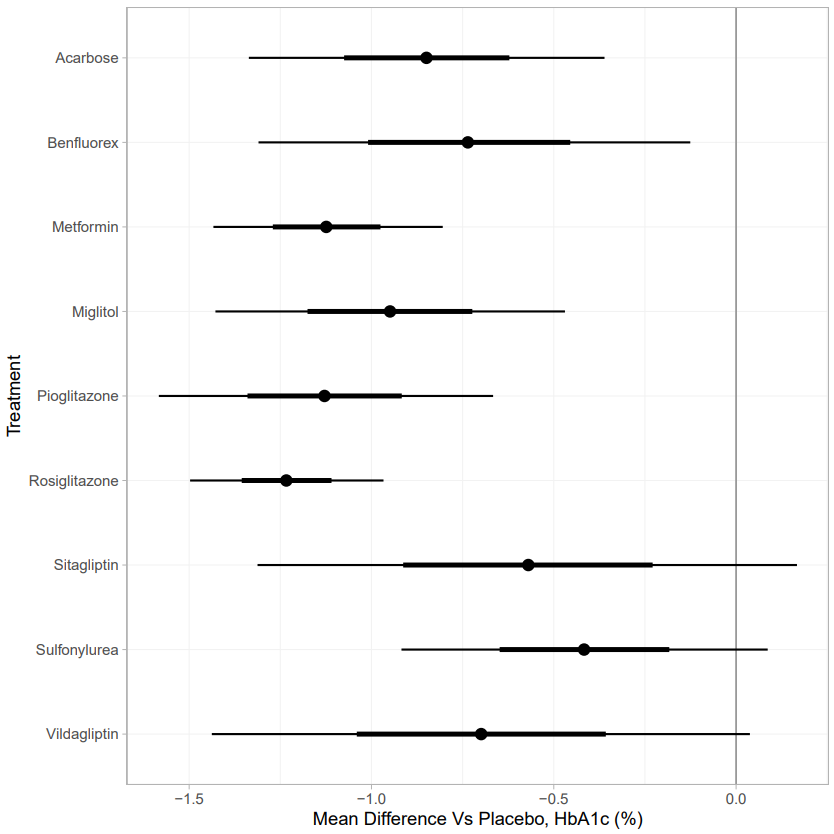


Supplementary Figure 5. Diabetes NMA: MID-adjusted probability j^th^ best (MID=0.3), where 1 is the best rank


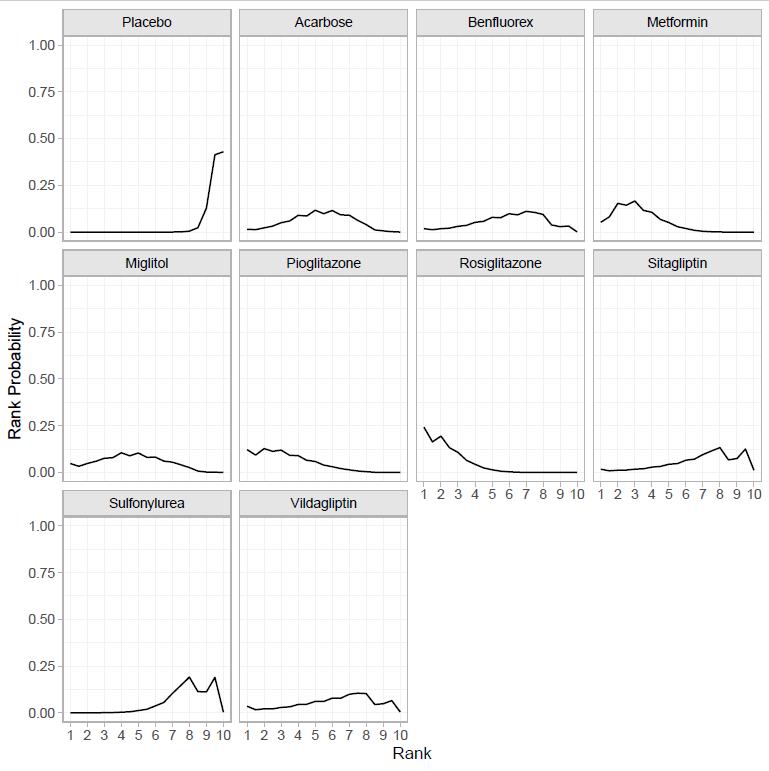


Supplementary Figure 6. Diabetes NMA: MID-adjusted cumulative probability j^th^ best (MID=0.3), where 1 is the best rank


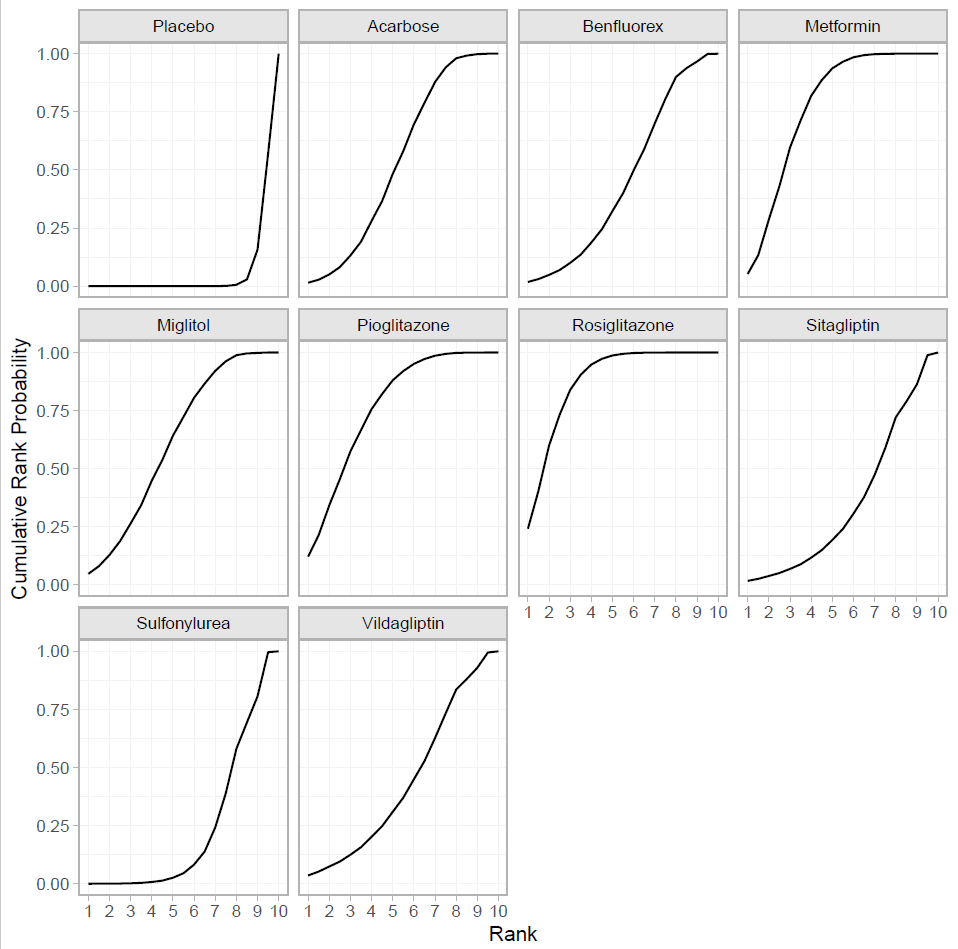

Supplement: Supplementary file 2 — Supplementary Material 2. [file 12874_2025_2499_MOESM2_ESM.docx]
